# Accessory spleen torsion: a hidden etiology of acute abdominal emergency

**DOI:** 10.1093/bjrcr/uaaf035

**Published:** 2025-07-16

**Authors:** Lucía Sanabria Greciano, Ana Fernández Alfonso, Begoña Peinado Iribar, Raquel Cano Alonso, Ana Álvarez Vázquez, Vicente Martínez de Vega Fernández

**Affiliations:** Radiology Department, Hospital Universitario Quirón Salud Madrid, 28223 Pozuelo de Alarcón, Madrid, Spain; Radiology Department, Hospital Universitario Quirón Salud Madrid, 28223 Pozuelo de Alarcón, Madrid, Spain; General Surgery Department, Hospital Universitario Quirón Salud Madrid, 28223 Pozuelo de Alarcón, Madrid, Spain; Radiology Department, Hospital Universitario Quirón Salud Madrid, 28223 Pozuelo de Alarcón, Madrid, Spain; Radiology Department, Hospital Universitario Quirón Salud Madrid, 28223 Pozuelo de Alarcón, Madrid, Spain; Radiology Department, Hospital Universitario Quirón Salud Madrid, 28223 Pozuelo de Alarcón, Madrid, Spain

**Keywords:** accessory spleen, splenectomy, laparoscopy, splenic infarction, torsion, congestion, splenosis

## Abstract

Accessory spleen torsion is a rare but important cause of acute abdominal pain, often presenting with non-specific symptoms that overlap with more common abdominal pathologies. This case report discusses a 19-year-old female who presented with left-sided flank pain and leucocytosis. Imaging with abdominal CT and MRI revealed a well-defined lesion near the spleen and kidney, with mild vascular engorgement and surrounding inflammation. While these findings raised suspicion for accessory spleen torsion, the diagnosis was not immediately clear. The lesion’s location, vascular congestion, and absence of typical characteristics for other pathologies, such as haematomas, abscesses, mesothelial cysts, or lymphangiomas pointed towards torsion, but confirmation required surgical intervention. During laparoscopic exploration, a 5 cm accessory spleen with ischaemic changes due to torsion of its pedicle was identified and successfully removed without complications. Accessory spleens, present in 10%-30% of the population, are usually asymptomatic but can become problematic if torsion, rupture, or infarction occurs. Imaging plays a critical role in identifying torsion, with CT and MRI revealing the characteristic “whirlpool sign” and vascular congestion. Early recognition is crucial to prevent complications such as necrosis and rupture, and surgical intervention, typically laparoscopic splenectomy, is the treatment of choice. This case highlights the importance of considering accessory spleen torsion in the differential diagnosis of acute abdominal pain, particularly in young patients with non-specific symptoms. Awareness of this condition can improve early diagnosis and outcomes, preventing severe consequences.

## Clinical presentation

A 19-year-old female presented to the emergency department with a few hours of left-sided flank pain. She denied nausea, vomiting, fever, changes in bowel movements, or urinary tract symptoms.

On physical examination, the abdomen was soft, non-distended, and tender to palpation in the left upper quadrant. Bowel sounds were present. There were no signs of peritoneal irritation, and physical examination findings including the Rovsing, Murphy, and Blumberg tests were negative. Laboratory results showed leucocytosis.

Referral to the gynaecology department was requested from the emergency room, where acute gynaecological pathology was ruled out upon evaluation.

## Imaging findings

**Figure 1.**
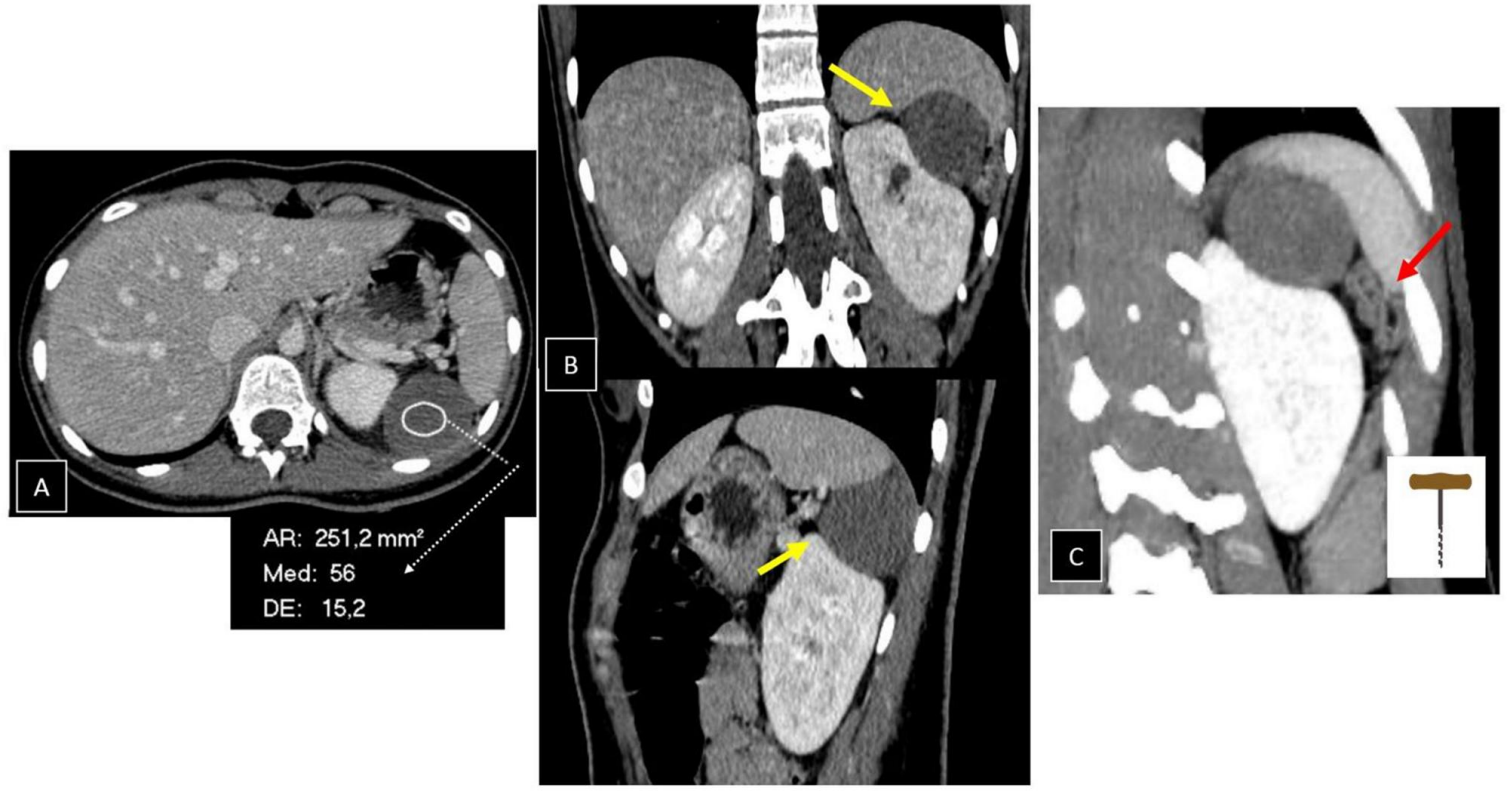
A contrast-enhanced abdominal CT in the venous phase shows a well-defined, likely unilocular lesion measuring 43 × 46 mm, located under the spleen, with a density range of 40-60 Hounsfield units. (A) The lesion mildly indents the lower border of the spleen and the upper-lateral aspect of the left kidney (yellow arrows), without clear organ dependence (B). On the right (C), an oblique coronal section is shown, where the tortuous dilation of the left gastroepiploic vessels of the twisted pedicle is visible, resembling a corkscrew shape (red arrow). No indications of subacute haemorrhage are observed.

A contrast-enhanced abdominal CT scan was performed in the venous phase, revealing a well-defined, apparently unilocular lesion measuring 43 × 46 mm, located immediately caudal to the spleen, with a density range of 40-60 Hounsfield units. The lesion slightly impressed upon the inferior border of the spleen and the superior-external contour of the left kidney, with no clear organ dependence and mild engorgement of the surrounding vessels. No haematocrit level was observed to suggest subacute haemorrhage. The appendix appeared normal, and no retroperitoneal or mesenteric lymphadenopathies were identified (Figure 1).

Initially, the case was focused on a possible mesothelial cyst/lymphangioma with signs of inflammation and no evidence of haemorrhage, and the patient was admitted under the care of the general surgery team for observation.

At the 24-h follow-up, laboratory results showed a worsening in the patient’s condition. The C-reactive protein increased from 6.70 to 13.20 (normal range: 0-0.5), and the leucocyte count rose from 12 to 13.47 × 10^3^/µL (normal range: 3.5-11). Due to the persistence of pain, the onset of nausea, and these worsening laboratory results, an abdominal MRI was ordered for further evaluation.

**Figure 2.**
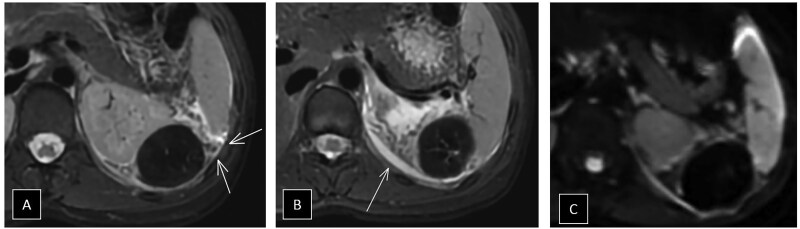
In the T2 axial images, the lesion appears distinctly hypointense, with a thickened and twisted vascular pedicle visible around it (A), as well as moderate locoregional inflammatory changes with a wedge-shaped perisplenic fluid collection (B). Additionally, the lesion did not show restriction of diffusion (C).

**Figure 3.**
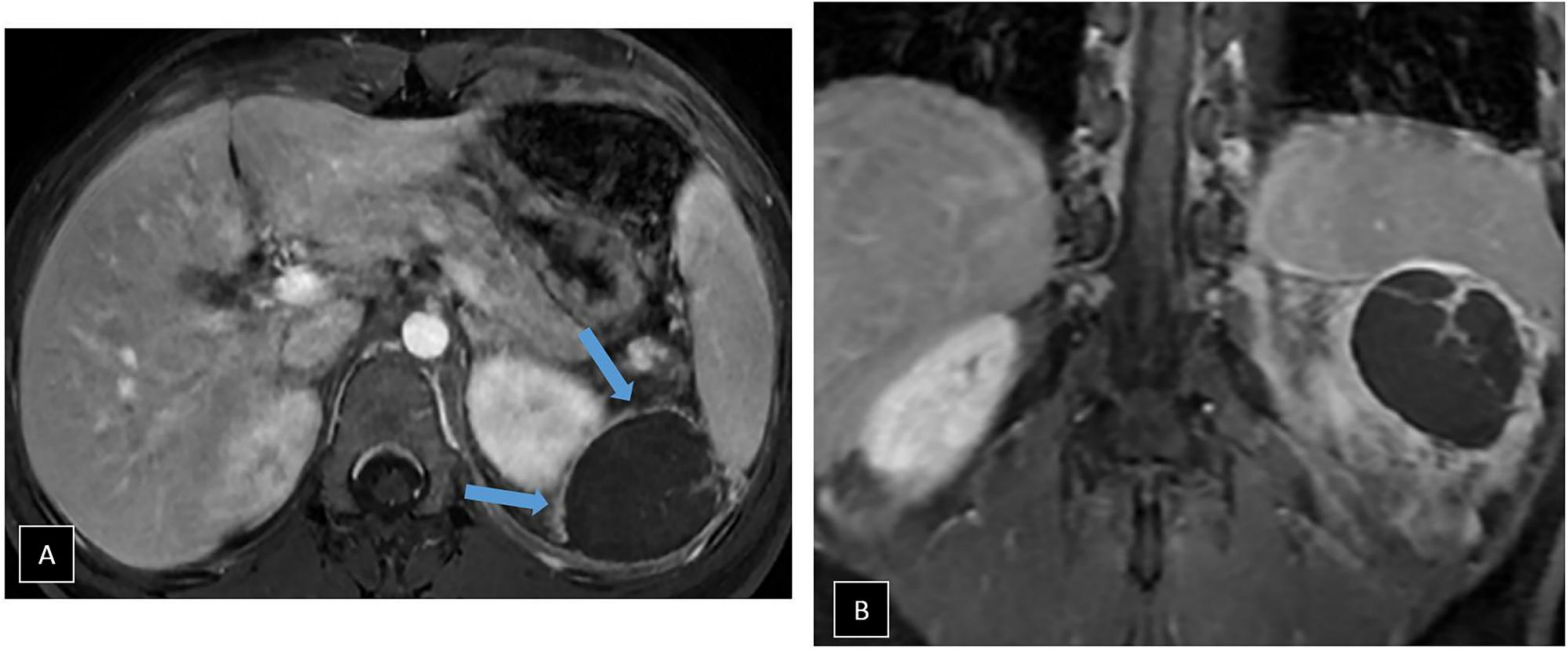
In the dynamic contrast-enhanced sequence (A), there was no enhancement of the lesion, except for a thin peripheral capsule (blue arrows). The post-contrast coronal image (B) showed persistence of the capsular enhancement in the late phases.

The contrast-enhanced abdominal MRI showed that the lesion was markedly hypointense on T2-weighted sequences (Figure 2A). Significant locoregional inflammatory changes were noted, with free perisplenic fluid and a mild left pleural effusion (Figure 2B). There was no restriction of diffusion (Figure 2C). Additionally, it was associated with engorged, tortuous vascular structures along its lateral margin, extending slightly down the flank. In the dynamic contrast-enhanced sequences (Figure 3), there was no enhancement of the lesion except for a thin peripheral capsule.

## Differential diagnosis

Given the findings described in the previous imaging studies (contrast-enhanced abdominal CT and contrast-enhanced abdominal MRI), a haematoma, an abscess, a mesothelial cyst, a lymphangioma with proteinaceus content and an accessory spleen torsion may be considered in the differential diagnosis^1^ (Table 1).

**Table 1.** Summary of differential diagnoses for the lesion in this case based on clinical and imaging findings.

Diagnosis	Imaging findings	Supporting/excluding features
Perisplenic haematoma	Well-defined slightly hyperdense lesion, no haematocrit level or subacute bleeding signs.	No recent trauma, absence of haematocrit levels, or bleeding signs make this diagnosis unlikely.
Perisplenic/retroperitoneal abscess	No diffusion restriction, lesion well defined.	Abscess typically presents with diffusion restriction; absence of this sign excludes this diagnosis.
Mesothelial cyst	Unilocular, non-enhancing lesion.	Located between spleen and kidney, with vascular engorgement and inflammation, which is atypical for a mesothelial cyst (usually asymptomatic, no inflammation).
Lymphangioma	Unilocular cystic mass, no multilocularity or septa.	Lack of multilocularity or septa rules out lymphangioma; presence of inflammation decreases likelihood.
Primary peritoneal tumour	Unilocular, non-enhancing lesion with no enhancement or diffusion restriction.	No peritoneal involvement or lymphadenopathy, and lack of typical enhancement or diffusion restriction make this diagnosis less likely.
Accessory spleen torsion	Well-defined lesion, vascular engorgement, no clear organ dependence, significant locoregional inflammation with perisplenic fluid.	Strong support from clinical presentation (left upper quadrant pain, leucocytosis) and imaging findings, particularly the engorged vascular pedicle. The absence of clear organ dependence strongly suggests torsion.

The table includes key characteristics of each diagnosis and the factors supporting or excluding its likelihood.

- *Perisplenic haematoma*: A well-defined, slightly hyperdense lesion could be consistent with a haematoma. However, the absence of haematocrit levels and subacute bleeding signs, as well as the lack of recent trauma, makes this diagnosis less likely.^2^
- *Perisplenic or retroperitoneal abscess*: While an abscess could present with a similar well-defined lesion, the lack of diffusion restriction, typically seen in abscesses, makes it unlikely.^3^
- *Mesothelial cyst*: Although the lesion’s unilocular and non-enhancing features could resemble a mesothelial cyst, the location between the spleen and kidney, along with vascular engorgement and significant inflammation, raises doubt. Mesothelial cysts are typically asymptomatic with no inflammatory signs.^4^
- *Lymphangioma*: A lymphangioma can present as a unilocular cystic mass, but its lack of multilocularity or septa, rules out this possibility. The presence of locoregional inflammation further decreases the likelihood.^4^
- *Primary peritoneal tumour*: The lesion’s unilocular shape and lack of typical features for primary peritoneal tumours (such as heterogeneous enhancement or diffusion restriction) make this diagnosis less likely. Furthermore, the absence of broad peritoneal involvement or lymphadenopathy further excludes this option.^5^
- *Accessory spleen torsion*: The diagnosis of accessory spleen torsion is strongly supported by the clinical presentation of left upper quadrant pain and leucocytosis, along with imaging findings of a well-defined lesion located between the spleen and kidney. Of particular importance is the engorgement of the vascular pedicle, which stands out as a key diagnostic feature. In addition to this, the absence of clear organ dependence and significant locoregional inflammation with perisplenic fluid, strongly suggests torsion. These findings, along with the acute pain, make accessory spleen torsion a highly probable diagnosis.^6^

Despite the different possibilities considered, the radiologist ultimately concluded that accessory spleen torsion was the most likely diagnosis. The combination of the clinical presentation, imaging findings, especially the vascular pedicle engorgement, and the absence of features typical of other conditions strongly supported this diagnosis.

## Treatment

**Figure 4.**
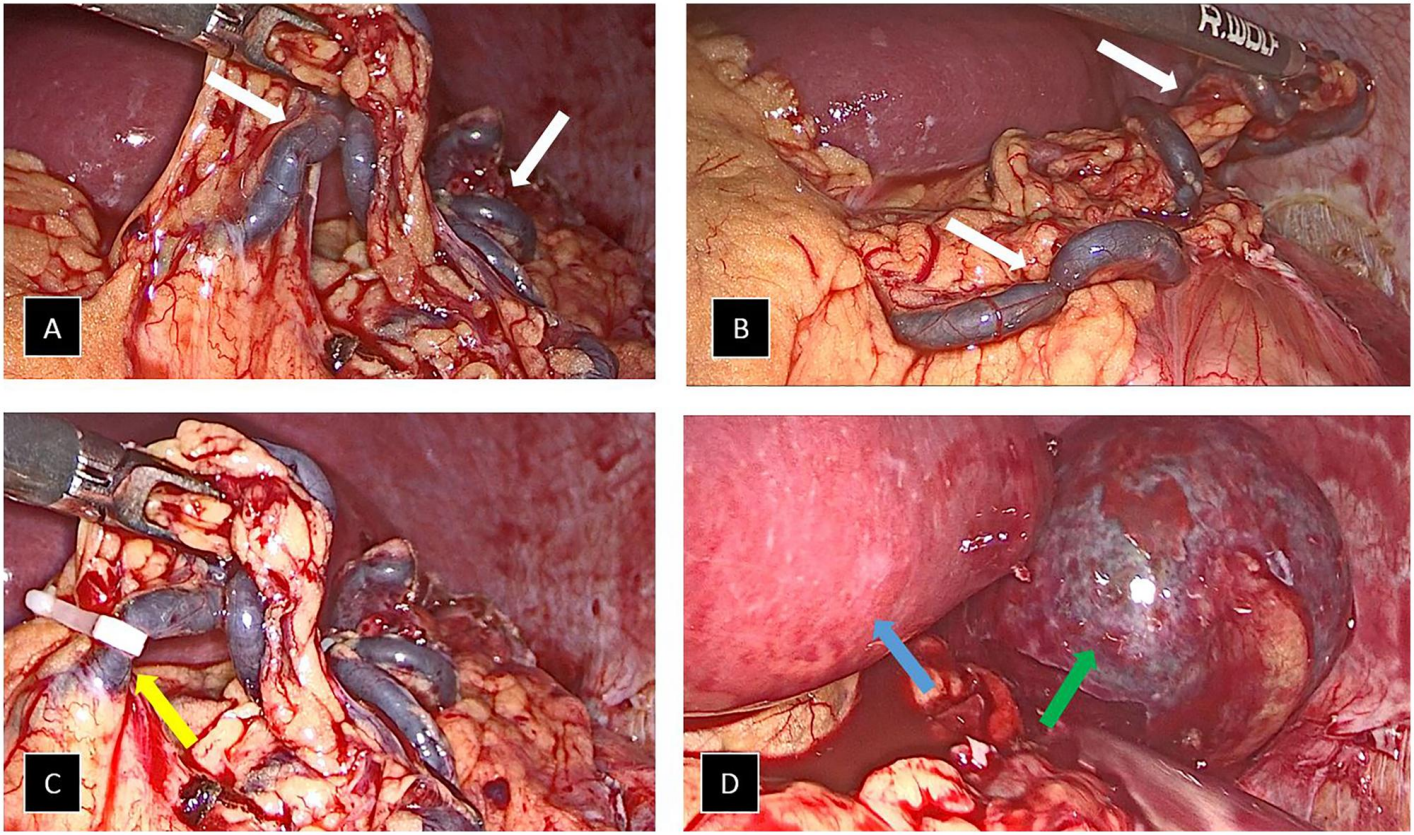
Intraoperative images of laparoscopic splenectomy showing the pedicle of the accessory spleen, dependent on the left gastroepiploic vessels, which appears thickened with signs of venous congestion and tortuosity (white arrows in images A and B). In image C, the clipping of the vessels of the pedicle with Hemolocks is identified (yellow arrow). Finally, in image D, the accessory spleen (green arrow) is identified, positioned laterally to the normal spleen (blue arrow), after being freed from inflammatory adhesions and prior to its removal in the protective bag.

Based on the MRI findings indicating the possibility of accessory spleen torsion, a laparoscopic procedure was performed. During the surgery, a 5 cm accessory spleen was identified in the left hypochondrium, with signs of ischaemia due to torsion of a dominant pedicle originating from the left gastroepiploic vessels (Figure 4). A splenectomy of the accessory spleen was successfully carried out without complications.

## Outcome and follow-up

The tissue sample obtained during surgery was sent to pathology for evaluation. The histological analysis revealed findings consistent with an accessory spleen, with no evidence of tumour cells or any other abnormal findings.

The patient was discharged from the hospital 48 h after surgery, without any complications in the postoperative period.

## Discussion

Accessory spleens, also known as splenules, are present in a significant portion of the population, with autopsy studies revealing an occurrence rate of 10%-30%. These are congenital and develop due to the incomplete fusion of splenic tissue buds during the fifth week of embryonic development.^7^

Each accessory spleen has its own blood supply, typically derived from a branch of the splenic artery, distinguishing them from splenosis. Unlike accessory spleens, splenosis is an acquired condition that typically occurs after splenectomy or splenic rupture, where fragments of non-encapsulated splenic tissue spread within the peritoneal cavity and are nourished by new blood vessels forming at the implantation site.^7^

The typical location of the accessory spleens is usually posteromedial to the spleen; anterolateral to the upper pole of the left kidney; and lateral, posterior, and cranial to the pancreas tail, although they may also be found in the gastrocolic ligament, mesocolon, pancreas, and even in some rare cases in the mediastinum.^8^

When identified, accessory spleens are typically asymptomatic, but their distinctive imaging features help differentiate them from other abdominal masses. Recognizing and properly identifying accessory spleens is important for several reasons. They can sometimes be confused with enlarged lymph nodes or masses in neighbouring organs, such as the pancreas, kidneys, or adrenal glands. Additionally, accessory spleens can occasionally lead to symptoms like twisting, rupture, bleeding, or cyst development. Finally, surgeons must be aware of their presence when aiming to excise all functional splenic tissue, particularly in patients undergoing treatment for haematologic conditions.^8^

When not complicated, an accessory spleen typically present on CT as well-defined, round masses, smaller than 2 cm that enhance homogeneously after contrast administration, showing a similar enhancement pattern to the spleen.^8^

However, when accessory spleen torsion occurs, the twisted pedicle obstructs venous and lymphatic outflow in the early stages, leading to swelling, congestion, and interstitial oedema. As the condition progresses, reduced arterial inflow may result in ischaemia, infarction, and inflammation of the affected tissues.^9^

Although ultrasound was not performed initially in our case due to unavailability at the time, it can sometimes be helpful as an initial diagnostic tool, revealing a hypoechoic, well-encapsulated oval mass with reduced blood flow on colour Doppler, which suggests oedema and necrosis.^9^

Imaging plays a critical role in diagnosing torsion, with certain CT features being key indicators. The “whirlpool sign” is a hallmark of torsion, where the twisted vascular pedicle appears as a spiral or swirling pattern on imaging, strongly indicating torsion.

In addition, affected organs often show enlargement due to venous congestion, and there may be signs of haemorrhagic infarction as tissue perfusion declines. Contrast-enhanced imaging typically reveals reduced enhancement in the later stages, reflecting compromised blood flow. Additionally, surrounding fat may show increased stranding or oedema, further highlighting the extent of inflammation. Despite poor internal enhancement, some structures, like the capsule, may still exhibit contrast enhancement due to preserved blood supply to the outer layers. These imaging findings are crucial for early diagnosis and management of torsion, preventing severe complications like organ necrosis or rupture.^9^

The gold standard for treating accessory spleen torsion is surgical intervention, specifically laparoscopic splenectomy. This approach is recommended for most cases, as it provides definitive resolution of the condition. The free nature of the accessory spleen allows for easy dissection, making single-port or single-port plus one laparoscopic techniques sufficient. However, in certain cases, particularly in the paediatric population with no clear signs of complete torsion and minimal symptoms, conservative management may be considered. This can include analgesics and antibiotics, with careful monitoring for any signs of re-torsion. In these cases, close follow-up is essential to ensure prompt intervention if symptoms persist or complications arise.^10^

## Conclusion

Accessory spleen torsion is a rare cause of acute abdominal pain that can mimic other conditions. Imaging findings, particularly the “whirlpool sign” on CT and MRI, are key to early diagnosis, suggesting a twisted vascular pedicle with associated vascular congestion and inflammation. However, preoperative diagnosis remains difficult, and definitive confirmation typically requires surgical exploration. Early recognition and prompt surgical intervention, typically through laparoscopic splenectomy, are essential to prevent severe complications and improve patient outcomes, particularly in young patients with unexplained left-sided abdominal pain, non-specific symptoms, and worsening laboratory values.

## Learning points

- Accessory spleen torsion is a rare but important cause of acute abdominal pain, particularly in paediatric patients and young individuals. It typically presents with left-sided flank pain and leucocytosis, but its symptoms can overlap with other conditions, making pre-surgical diagnosis challenging.
- Imaging plays a crucial role in detecting torsion, with CT and MRI often showing key features such as the “whirlpool sign,” vascular engorgement of the twisted pedicle and surrounding inflammation. While these findings help raise suspicion, torsion can’t always be confirmed without surgical intervention.
- Surgical intervention is usually required for both confirmation and treatment. While imaging can strongly suggest torsion, excision of the accessory spleen is typically necessary for a definitive diagnosis. Early surgery also helps prevent complications like ischaemia, infarction, and rupture, leading to favourable outcomes. Conservative treatment may be considered for mild cases without complications, but close monitoring is essential to assess the risk of re-torsion.
